# An Overview of Electrochemical Sensors Based on Transition Metal Carbides and Oxides: Synthesis and Applications

**DOI:** 10.3390/mi15010042

**Published:** 2023-12-24

**Authors:** Amirarsalan Mashhadian, Ruda Jian, Siyu Tian, Shiwen Wu, Guoping Xiong

**Affiliations:** Department of Mechanical Engineering, The University of Texas at Dallas, 800 W Campbell Rd., Richardson, TX 75080, USA

**Keywords:** transition metal carbides, transition metal oxides, electrochemical sensors, two-dimensional materials

## Abstract

Sensors play vital roles in industry and healthcare due to the significance of controlling the presence of different substances in industrial processes, human organs, and the environment. Electrochemical sensors have gained more attention recently than conventional sensors, including optical fibers, chromatography devices, and chemiresistors, due to their better versatility, higher sensitivity and selectivity, and lower complexity. Herein, we review transition metal carbides (TMCs) and transition metal oxides (TMOs) as outstanding materials for electrochemical sensors. We navigate through the fabrication processes of TMCs and TMOs and reveal the relationships among their synthesis processes, morphological structures, and sensing performance. The state-of-the-art biological, gas, and hydrogen peroxide electrochemical sensors based on TMCs and TMOs are reviewed, and potential challenges in the field are suggested. This review can help others to understand recent advancements in electrochemical sensors based on transition metal oxides and carbides.

## 1. Introduction

Sensors play a crucial role in monitoring the health of our environment [1]. They can be used in applications such as industrial production, food safety, biomedicine, and environmental monitoring [2,3,4]. Spectroscopic and chromatographic approaches are conventional techniques in sensing applications. Nevertheless, they suffer from limited selectivity and sensitivity, complex operational procedures (e.g., extraction steps), constrained adaptability, sample manipulations, and unsuitability for solutions that are colorful or turbid [5,6]. Electrochemical sensors have received significant attention due to their cost effectiveness, high selectivity and sensitivity, and reasonable versatility [4]. Among the materials used for electrochemical sensing, transition metal oxides (TMOs) and transition metal carbides (TMCs) are extensively employed because of their simple structural designs and fabrication processes [7,8].

TMO nanomaterials with spherical, nanowire, and nanosheet shapes have shown many desirable properties, including good electronic tunability, varied functionalization, exceptional thermal and chemical stability, non-toxicity, and different kinds of bandgaps, for a variety of electrochemical sensing applications, such as gas sensing, biological sensing, and environmental monitoring [7,9,10,11]. The most frequently presented configurations of TMOs in the literature are multi-layer, single-planar, and superlattice structures, which can be used to fabricate electrochemical sensors with different selectivity, sensitivity, and stability [12]. Several methods have been introduced to prepare TMOs, but chemical vapor deposition (CVD) and hydrothermal techniques are among the most reported ones [13]. TMCs are one type of MXene, a new two-dimensional (2D) material that has the general format of M*_n_*_+1_X*_n_*T*_x_*, where M, N, and T_x_ account for transition metal, carbon (C) or nitrogen (N), and surface functional groups (such as OH^−^ and F^−^), respectively [14]. Thanks to the unique features of TMCs, including large surface area, metallic properties, surface chemistry tunability, and high electrical conductivity, they are promising candidates for the detection of biological and environmental species [8]. Unsaturated *d* orbitals of transition metals used in both TMCs and TMOs contribute to the band structure of the prepared materials, leading to specific features like superconductivity [15,16]. Similar to TMOs, the fabrication of TMCs is not confined to standard techniques (i.e., CVD-assisted and hydrothermal), and researchers have discovered additional synthesis routes, such as bottom-up chemical reaction and laser ablation [17].

Electrochemical sensors consist of at least two electrodes, including a working electrode and a counter electrode, to make a close electrical circuit and a transducer, which is essential for charge transport [18]. However, the charge transport in the targeted substance can be electronic, ionic, or a combination of the two. In this configuration, a chemical interaction between the working electrode surface and the targeted analyte may result in charge or ion transfer. The preconcentration technique has been implemented in electrochemical sensors to help accumulation or concentration of the desired analyte at the surface of the electrode prior to the electrochemical measurement [19]. The preconcentration method includes a variety of processes, but two primary ones for enhancing the performance of electrochemical sensors are electrochemical deposition and adsorption [20]. Electrochemical deposition is the process by which an analyte undergoes either reduction or oxidation and is then deposited onto the surface of an electrode [21]. Furthermore, adsorption processes include the binding of analyte molecules to the surface of the sensor via electrostatic interaction or binding mechanisms, allowing for the detection of a broad spectrum of analytes [22]. After all, the magnitude of the electric current or voltage resulting from charge or ion transfer between the analyte and the working electrode suggests the concentration of the targeted samples. Cyclic voltammetry (CV), linear sweep voltammetry (LSV), square wave voltammetry (SWV), and differential pulse voltammetry (DPV) are different voltammetry techniques that have been commonly employed to determine the electrochemical responses of the targeted substance [6,7].

Tran et al. [23] discussed the MXene fabrication methods and applications in sensors. In particular, they gave an overview of how MXene works in biological electrochemical sensors and showed how well the electrochemical sensors perform. Recently, many papers surveyed TMOs in sensing applications [24,25,26,27,28]. For instance, TMO electrochemical sensors in healthcare applications have been reviewed in prior work [28], which highlighted that TMOs in biological electrochemical sensors provide significant benefits in terms of durability and sensitivity for detecting cancer biomarkers, but also acknowledged the presence of some limitations. Detailed comparison of the use of TMOs and TMCs in electrochemical sensors would benefit the field. This review paper highlights recent advancements in electrochemical sensors based on TMCs and TMOs and summarizes the enormous efforts that have been made toward the design strategies of these materials. The synthesis methods and resulting morphologies of TMOs and TMCs are discussed. Then, applications of TMOs and TMCs in gas sensors, biosensors, and hydrogen peroxide sensors are presented, emphasizing the sensitivity and limit of detection (LOD) of these sensors. Thereafter, future potential challenges in the field are also stated. 

## 2. Syntheses of Transition Metal Carbides and Transition Metal Oxides

### 2.1. Introduction

TMOs and TMCs are promising nanomaterials for applications ranging from energy storage and electronics to biosensing because of their unique properties, including high thermal stability, high thermal/electrical conductivity, outstanding chemical stability, and excellent mechanical performance [29,30]. For instance, depending on their compositions and crystal structures, the electronic behavior (e.g., band gap) of TMOs and TMCs can be precisely tuned from insulating to metallic, making them pivotal in developing electronic and optoelectronic devices [31]. In general, TMOs and TMCs are formed via the reactions of Group 4–10 transition metals with oxygen and carbon atoms, respectively. The oxygen or carbon atoms can form various types of bonds (e.g., covalent, ionic, metallic) with transition metals, leading to an extensive family of binary, ternary, and complex oxides or carbides in face-centered cubic (FCC), hexagon-closed packed (HCP), and simple hexagonal structures [32]. In addition, the unique physicochemical properties of TMOs and TMCs can also be tuned via their dimension, morphology, and defects, which are highly dependent on synthetic methods.

To date, many strategies have been proposed to synthesize TMOs and TMCs with different characteristics and functionalities. In general, these synthetic methods can be categorized into top-down exfoliation and bottom-up self-assembling categories [12]. Top-down exfoliation involves producing 2D TMO and TMC nanosheets from their bulk materials via mechanical exfoliation, liquid-phase exfoliation, and electrochemical exfoliation. During the top-down exfoliation process, external forces are applied to separate adjacent layers by overcoming the van der Waals interactions, yielding high-quality TMO and TMC nanosheets with high crystallinity. In comparison, the bottom-up synthesis of TMOs and TMCs can be achieved through the decomposition of specific precursors and the sequential self-assembling of resultant active species. These bottom-up methods, including chemical vapor deposition (CVD), electrodeposition, and hydro-/solvo-thermal, have been widely employed to synthesize TMOs and TMCs with well-controlled physicochemical properties. In this section, we highlight the state-of-the-art research on TMOs and TMCs, with a focus on their synthetic methods and material nanostructures. We aim to provide useful information for the development of sensors based on a profound understanding of the material–structure–performance relationships associated with emerging TMOs and TMCs.

### 2.2. Hydrothermal Method

Hydrothermal synthesis is an adaptable method in materials science carried out in an aqueous environment at controlled temperatures and pressures [33]. Water reactants undertake reactions inside a high-pressure autoclave, which allows water to remain in the liquid form beyond its boiling point, which is perfect for controlling crystal growth. The nature of the aqueous solution allows for well-defined structure formation [34], and by modifying conditions, a variety of complicated nanostructures can be formed. Hydrothermal synthesis is distinguished by its versatility and precision in producing various and complex materials, especially for sensor electrodes containing TMOs and TMCs.

**Figure 1:**
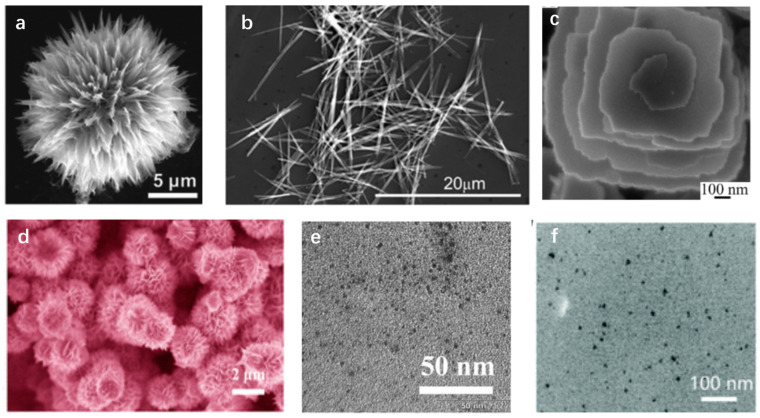
Optical images of TMCs and TMOs fabricated via hydrothermal methods. SEM images of (**a**) hierarchical flower-like WO_3_ [35], (**b**) MoO_3_ nanoribbons (reproduced with permission from Ref. [24]. Copyright 2019, American Chemical Society), (**c**) helical MoO_3_ nanostructures (reproduced with permission from Ref. [36]. Copyright 2006, American Chemical Society), (**d**) hierarchical SnO_2_ nanostructures (reproduced with permission from Ref. [37]. Copyright 2014, American Chemical Society). TEM images of (**e**) photoluminescence Ti_3_C_2_ QDs (reproduced with permission from Ref. [38]. Copyright 2017, Wiley), (**f**) surface-modified Ti_3_C_2_ QDs (reproduced with permission from Ref. [39]. Copyright 2017, Royal Society of Chemistry).

Xiao et al. [35] reported for the first time a method for fabricating hierarchical flower-like WO_3_ nanostructures constructed from needle-like single-crystalline nanosheets. By mixing tungstate powder with K_2_SO_4_ and subsequently acidifying the mixture using an HCl solution, a stable flower-like WO_3_ composed of relatively loosely packed nanosheets and with a diameter of 16 μm was obtained, as shown in Figure 1a. This hierarchical structure exhibits promising potential for sensor applications. To fabricate α-MoO_3_ nanoribbons, Kwak et al. [24] used molybdenum metal powder sealed in the autoclave after hydrogen peroxide treatment as a precursor, leading to nanoribbons with a thickness of approximately 10–12 nm and a lateral dimension of ∼200 nm, with morphology depicted in Figure 1b. Numerous prior studies have employed the hydrothermal method for the synthesis of MoO_3_, yielding distinct morphologies. For instance, Li et al. [36] successfully produced MoO_3_ with various shapes, including helical nanosheets, crosslike nanoflowers, and nanobelts, utilizing an environmentally sustainable chemical route on a large scale. Metallic molybdenum powder was used as a precursor, mixed with distilled water and hydrogen peroxide, and then transferred to an autoclave for a hydrothermal process at 180 °C for 2 to 12 h. Interestingly, some nanosheets tend to be twisted to form helical structures, as shown in Figure 1c. Different morphologies, ranging from helical nanosheets and crosslike nanoflowers to nanobelts, have been observed in various studies using the hydrothermal technique, demonstrating the flexibility of the hydrothermal method for fabricating TMOs.

Other explorations have been made into the field of TMO fabrication. For instance, Liu et al. [37] employed a hydrothermal method to prepare hierarchical SnO_2_ nanostructures (HTNs), for which the scanning electron microscope (SEM) image is shown in Figure 1d. By utilizing SnCl_2_·2H_2_O, C_6_H_5_Na_3_O_7_·2H_2_O, and ethanol–water solution, the HTNs were produced inside a Teflon-lined stainless-steel autoclave. The resultant samples exhibited a dense arrangement of hierarchical, flower-shaped structures composed of ultrathin nanosheets. The hydrothermal method is also the main route for the fabrication of TMCs. A distinctive approach to preparing photoluminescent Ti_3_C_2_ MXene-based quantum dots (MQDs) with impressive quantum yields of up to 10% was introduced by Xue and coworkers [38]. They obtained Ti_3_C_2_ (Figure 1e) by selectively etching the Al layer in the Ti_3_AlC_2_ MAX phase using a 48% hydrofluoric (HF) solution. Such breakthroughs in the synthesis of photoluminescent MQDs highlight their potential in diversifying the applications of MXenes. In a related research study, Chen et al. [39] presented a novel category of surface-modified Ti_3_C_2_ quantum dots (QDs) utilizing a combination of sonication cutting and a hydrothermal method to prepare TMCs (Figure 1f). Although hydrothermal methods have been extensively used for TMOs and TMCs fabrication, they face severe environmental issues due to the use of hazardous materials (i.e., HF, chlorine gas, etc.) during the fabrication process. Furthermore, the protracted nature of certain processes and the lack of exact control over crystal growth in hydrothermal methods have prompted researchers to explore other methodologies.

Controlling the shape of TMOs during fabrication without compromising other parameters is crucial and significantly affects their sensing performance. For instance, Liu et al. [40] synthesized cobalt oxide (Co_3_O_4_) nanocubes and nanospheres utilizing the hydrothermal method. An attempt was made to produce Co_3_O_4_ nanocubes and nanospheres using a comparable method. In a similar study, Roy et al. [41] observed variations in the morphology of Co_3_O_4_ by adjusting the period of heating time in a comparable investigation. The Co(NO_3_)_2_·6H_2_O, urea (CO(NH_2_)_2_), and water combination was subjected to a temperature of 150 °C for 2, 5, 12, and 24 h. The analysis revealed that the samples treated for 2 and 5 h exhibited a nanorod morphology, whereas those treated for 12 and 24 h showed a nanosheet morphology. The aforementioned papers may provide valuable insights into the design of TMOs by controlling their morphology for enhanced sensing performance.

### 2.3. Chemical Vapor Deposition Method

CVD is known for its ability to manufacture high-purity, high-performance solid materials for applications ranging from thin films to optical fibers and electronics [42]. Thermal CVD involves the introduction of gaseous or liquid precursors into a reactor under high temperatures and controlled pressure, producing reactive species due to the decomposition of the precursors. The as-formed species interact with the substrate, forming a solid film on the substrate. The versatility of thermal CVD ensures tunable material properties by varying precursors, reactor conditions, and substrates. Atmospheric pressure CVD (APCVD) [43], low-pressure CVD (LPCVD) [44], plasma-enhanced CVD (PECVD) [45], and metal–organic CVD (MOCVD) [46] are the other types of CVD used for TMOs and TMCs fabrication. In particular, the PECVD method, due to its tendency for lower temperature depositions, is suitable for the growth of nanomaterials with heat-sensitive substrates [47,48].

**Figure 2:**
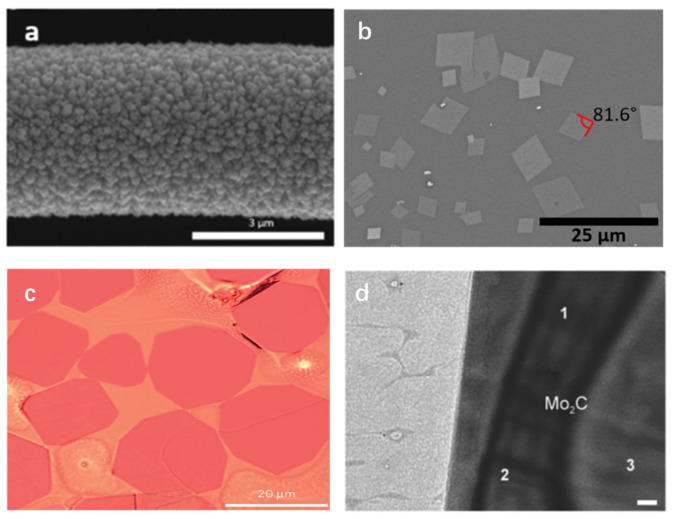
Optical images of TMCs and TMOs fabricated via thermal CVD. SEM images of (**a**) WO_3_ deposited on GCW (reproduced with permission from Ref. [49]. Copyright 2020, Elsevier), (**b**) MoO_3_ nanosheets (reproduced with permission from Ref. [50]. Copyright 2020, Elsevier), (**c**) ultrathin α-Mo_2_C crystals on a Cu/Mo substrate (reproduced with permission from Ref. [51]. Copyright 2020, IOP Science), (**d**) TEM image of the Mo_2_C on graphene film (reproduced with permission from Ref. [52]. Copyright 2017, Wiley).

In a pioneering study, Cisquella-Serra et al. [49] presented the localized CVD of WO_3-x_ on a suspended glassy carbon wire (GCW). During this process, a 1:1 mixture of acetone/ dimethylformamide was heated under reduced pressure and evaporated before being transferred to the CVD chamber. As depicted in Figure 2a, they successfully fabricated WO_3−x_ coatings with thicknesses between 71 nm and 1.4 μm on glassy carbon wires with diameters ranging from 780 nm to 2.95 μm. This advancement has paved the way for the development of diverse GCW-based sensors. Furthermore, the CVD method has proven effective for the synthesis of other TMO materials. Utilizing the CVD technique, Saenz et al. [50] successfully synthesized MoO_3_ nanosheets through the reactions between MoO_3_ and sulfur (S) powder precursors, employing N_2_ as the carrier gas. The precursors underwent heating, reaching peak temperatures of approximately 850 °C and 174 °C for MoO_3_ and S, respectively. As shown in the SEM (Figure 2b), triangular and hexagonal nanosheets with a thickness of approximately 5.32 nm were fabricated. TMCs can also be synthesized using the conventional thermal CVD method. 

In a groundbreaking study, the large-area, high-quality 2D ultrathin α-Mo_2_C crystals were fabricated by Xu and colleagues [53] employing the thermal CVD method. Methane was utilized as the carbon source, and a Cu foil layered over a Mo foil was used as a substrate in the deposition process at a temperature of approximately 1085 °C. These high-quality 2D ultrathin α-Mo_2_C crystals exhibited nanoscale thickness while possessing lateral dimensions over 100 μm. Impressively, the synthesized TMCs showed remarkable stability across a variety of liquids and elevated temperatures, which underscored their potential for diverse applications, including sensing. In a similar study, Geng et al. [51] illustrated the production of ultrathin Mo_2_C crystals (Figure 2c) on a liquid Cu surface via CVD. This technique allowed them to control the morphology of the crystals by modulating the carbon supersaturation. Furthermore, Geng et al. [52] introduced an alternative approach for the direct one-step synthesis of 2D Mo_2_C on graphene film, using molten copper-catalyzed CVD. The transmission electron microscopic (TEM) image of the 2D Mo_2_C is shown in Figure 2d. The manipulation of Cu and Mo (precursor) foils, along with varying quantities of H_2_ and CH_4_ gases, enables the regulation of the growth of either the heterostructure Mo_2_C/graphene or Mo_2_C material. Graphene passivation in this method prevented Mo and hydrocarbon interactions, shifting from the precipitation to diffusion-driven growth and enabling the precise control of nanoscale Mo_2_C growth.

### 2.4. Other Methods

**Figure 3:**
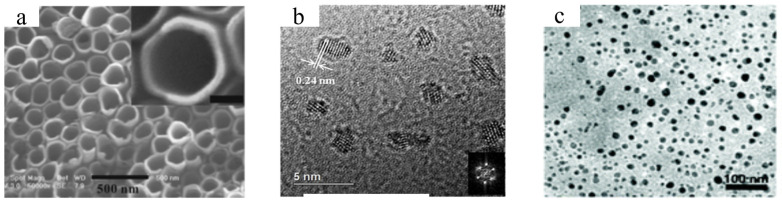
Optical images of TMCs and TMOs fabricated via different methods. (**a**) SEM images of TiO_2_/Au fabricated via rapid annealing method (reproduced with permission from Ref. [54], copyright 2016, American Chemical Society), (**b**) TEM image of Nb_2_C QDs (reproduced with permission from Ref. [56], copyright 2020, American Chemical Society), (**c**) SEM image of Ti_3_C_2_T_x_ produced via force-assisted liquid exfoliation (reproduced with permission from Ref. [57], copyright 2017, Royal Society of Chemistry).

While hydrothermal and CVD methods have been popularly adopted for the synthesis of TMOs and TMCs, a number of alternative fabrication techniques have emerged. In a research study by Hu and his team [54], TiO_2_/Au was fabricated by rapidly annealing anodized TiO_2_ nanotubes in argon and then electrodepositing Au nanoparticles. The morphology of as-prepared TiO_2_/Au is shown in Figure 3a. The C-doped TiO_2_ electrode was created by electrochemically anodizing titanium foil in an NH_4_F–ethylene glycol combination. After the anodization, the remaining ethylene glycol was used as a carbon source during argon annealing at 500 °C. Chekin et al. [55] presented a green approach for the synthesis of pure cobalt oxide (CoO) nanoparticles using gelatin in an aqueous medium. Cobalt nitrate was dissolved in deionized water and was mixed with gelatin before being agitated at 80 °C for 12 h to make resin. After cooling, it was treated in a furnace at 500 °C for 8 h, producing 25-nm CoO nanoparticles that are attractive for biological sensing applications due to their size and characteristics. 

Significant research efforts are being dedicated to improving the synthetic procedures for TMC materials such as TiO_2_, Ti_3_C_2_T_x_, Nb_2_C, and Mo_2_C to improve the performance of the sensors [11,58,59,60,61]. For instance, Yang and his group [56] reported a novel approach for manufacturing Nb_2_C QDs with pristine crystallographic structures of Nb_2_C MXene phases and surface oxygen-containing species, as shown in Figure 3b. HF was used to etch Nb_2_AlC powder, and Nb_2_C stacks were treated with tetrapropylammonium hydroxide and pulsed ultrasonication. After filtration and dialysis, freeze-drying produced Nb_2_C quantum dots with the MXene phase structure, ensuring chemical stability and biocompatibility. Yu et al. [57] developed a new mechanical force-assisted liquid exfoliation method to produce Ti_3_C_2_ MXene QDs with outstanding near-infrared (NIR) photothermal properties. This approach involved the sonication of bulk Ti_3_AlC_2_ in tetra-n-butylammonium hydroxide etching solution, resulting in isolated Ti_3_C_2_ nanosheets, as displayed in Figure 3c. 

Table 1 provides the advantages and drawbacks of the fabrication methods of TMOs and TMCs, including CVD, hydrothermal, controlled electrodeposition, and acid etching methods.

## 3. Sensing Applications

### 3.1. Transition Metal Carbides

TMCs have recently attracted significant attention in various applications, particularly in sensing, including gas sensing, biological sensing, and hydrogen peroxide detection [62,63]. Therefore, a comprehensive overview of materials based on TMCs and their related sensing performance is provided in this section.

#### 3.1.1. Gas Electrochemical Sensors

Sensing harmful gases is crucial owing to the potential damage that the inhalation of such gases may bring to human health. TMCs are emerging materials capable of detecting an array of gases, including toxic gases, because of their high surface functionality [64]. One of the first studies about TMC sensors was conducted by Yu et al. [65], who evaluated the effectiveness of monolayer Ti_2_CO_2_ for sensing multiple gases, like NH_3_, H_2_, CH_4_, CO, CO_2_, N_2_, NO_2_, and O_2_. They employed the first-principles calculation to investigate which gases could be adsorbed by the monolayer carbide. Their results demonstrated that Ti_2_CO_2_ is a promising candidate for sensing NH_3_ gases, and there is no sensitivity toward other gases. This study supported the findings of Lee et al. [66], who investigated Ti_3_C_2_T_x_ nanosheets for the susceptibility of detecting different volatile organic compound (VOC) gases. Among all the gases, NH_3_ received the highest response from the fabricated sensor grown on a flexible polyimide substrate. This study suggested that the sensing mechanism relies on the reaction of different gases with the functional groups on the surface of the sensor. For example, NH_3_ adsorbed on the surface of the Ti_3_C_2_T_x_ interacts with O and OH functional groups, as shown in Equations (1) and (2), respectively.

2NH_3_ + 3O^−^  →  N_2_ + 3H_2_O + 3e^−^ (1)

NH_3_ + OH^−^  →  NH_2_ + H_2_O + e^−^ (2)

In specific applications, such as those requiring the presence of hazardous gasses like ammonia, a higher level of sensitivity is necessary [67]. For instance, Kim et al. [68] designed a Ti_3_C_2_T_x_ sensor capable of sensing gases at concentrations as low as 50 parts per billion (ppb). The constructed sensor has two characteristics: (1) high conductivity and (2) a large number of adsorption sites, which resulted in minimal electrical noise and a strong signal. In another work, Lee and coworkers [69] reported that Ti_3_C_2_T_x_ is highly sensitive to NH_3_. Moreover, controlling the atomic structure of the MXene is beneficial to improving the detection of NH_3_ gas. To investigate this, Li et al. [70] conducted density functional theory (DFT) calculations to understand the effect of Ti-deficient in Ti_3_C_2_O_2_ 2D material on NH_3_ sensing. Their findings indicated that the presence of Ti-deficient can lead to the higher adsorption energy of NH_3_ and the current change. Consequently, precise modifications to the atomic structure could be beneficial for improving sensitivity.

Moreover, TMCs have also been fabricated for other types of gas detection. Yang et al. [71] investigated the potential of several TMCs (Sc_2_CO_2_, Ti_2_CO_2_, Zr_2_CO_2_, and Hf_2_CO_2_) for detecting NO and CO gases using DFT calculation. Their results indicate that Sc_2_CO_2_ is a good candidate for NO molecule detection since NO molecules chemically reacted with the sensor. In addition, they claimed that Mn dopant substantially increased the selectivity of the SC_2_CO_2_ toward CO molecules, opening a new window for the enhancement of CO sensitivity. In another study by Rathi et al. [72], highly conductive niobium carbide (Nb_2_CT_x_) was used as a sensor for NO_2_ gas. The obtained results show high sensitivity of the sensor to NO_2_ gas (0.543 ppm) that can be made more stable and efficient by adding surface surfactants like cetyltrimethylammonium bromide. 

In addition to toxic gases, explosive and flammable gases (usually non-polar) must be detected due to the financial and human life damage they may inflict. Non-polar gases are difficult to track because they are hardly adsorbed on the surface. To resolve this issue, Lee et al. [73] initiated research on the responsiveness of synthesized 2D vanadium carbides (V_2_CT_x_) to non-polar gases. The synthesized vanadium carbide showed a great response to both polar and non-polar gases, with a low detection limit of 25 and 2 parts per million (ppm) for methane and hydrogen, respectively.

#### 3.1.2. Biological Electrochemical Sensors

The conventional cancer detection methods are characterized by their high cost and time-consuming nature, potentially discouraging individuals from undergoing such examinations [74]. Biosensors, with their rapid and precise biomolecule detection capabilities, have broad applications in healthcare, particularly in cancer diagnosis, dramatically improving survivability and treatment success rates [75,76]. The high reactivity, cost effectiveness, and biodegradability of TMCs have opened a new window to early-stage cancer diagnosis [77]. 

**Figure 4:**
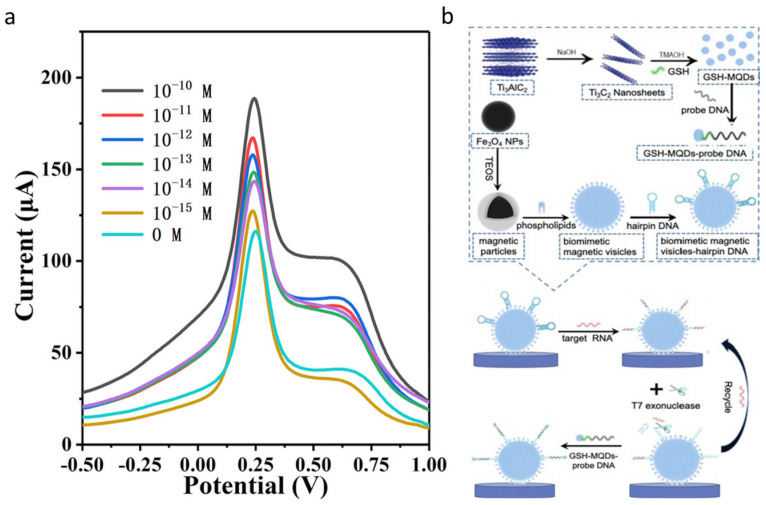
(**a**) DPV response of the MoS_2_/Ti_3_C_2_ at different concentrations of miRNA–182 (reproduced with permission from Ref. [78], copyright 2019, Elsevier), (**b**) schematic of fabrication procedure of an electrochemical sensing system based on MQDs and biomimic magnetics vesicles (reproduced with permission from Ref. [79], copyright 2022, Elsevier).

The identification of microRNA (miRNA) is one of the techniques utilized to ascertain the presence of cancer within the human body. miRNA-182 is associated with lung cancer, and it can play a role as a lung cancer biomarker. Liu et al. [78] synthesized an ultrasensitive sensor made of MoS_2_/Ti_3_C_2_ nanohybrid. They introduced a new label-free method for the fabrication of TMCs with abundant active sites and high conductivity. The adsorption of negatively charged miRNA to the surface caused a tangible current change in DPV current with linear range and LOD equal to 1 fM to 0.1 nM and 0.43 fM, respectively (Figure 4a). Another miRNA, miRNA-22, which is associated with breast cancer, was detected using TMC quantum dots (Figure 4b for mechanism) [79]. In the last study, glutathione was used in order to introduce the sulfhydryl group to the TMC atoms and consequently increase the stability of the material with nanostructure defect reduction, while the sensor exhibited an LOD as low as 10 fM.

The determination of distinct biomarkers, which are molecular compounds generated by malignant cells, is a widely recognized approach in the field of cancer detection. Carcinoembryonic antigen (CEA) is typically discovered in people with lung, colorectal, breast, and liver malignancies [80]. Identifying CEA in the human body indicates one of the aforementioned cancers; thus, sensing this biomarker is extremely vital. In order to detect CEA effectively, aminosilane-functionalized Ti_3_C_2_ has been synthesized by Kumar et al. [81]. The functionalized Ti_3_C_2_ nanosheets showed ultrasensitive behavior toward CEA with a sensitivity of 37.9 µA·ng^−1^·mL·cm^−2^ per decade.

Toluene has been identified as a biomarker for lung cancer, and thus the detection of toluene may serve as an indication of the existence of lung cancer [82]. A new 2D nanocomposite comprised of Pt/Ti_3_C_2_T_x_-carbon nanotube was synthesized and tested for quick diagnosis of lung cancer [83]. Also, this sensor offered a relatively low LOD of close to 2 ppm and a working temperature of around 150 °C, which is lower than similar reported sensors. To prove the effectiveness of TMCs in VOC detection, Reji et al. [84] employed DFT calculations to propose that SC_2_CO_2_ is sensitive to physiosorbed VOC such as toluene. In summary, TMCs have a wide range of applications in cancer diagnosis, exhibiting certain advantages over traditional cancer detection, such as rapid reaction and cost effectiveness.

#### 3.1.3. Hydrogen Peroxide Electrochemical Sensors

Hydrogen peroxide detection is important in a wide range of applications, such as human safety, medical diagnosis, and industrial process control [85]. Consequently, fabricating sensors that can provide acceptable sensitivity toward hydrogen peroxide is highly demanded. Annalakshmi and coworkers [86] fabricated a facile non-enzymatic hydrogen peroxide sensor made of cobalt nanoparticle (CoNP)-decorated tungsten carbide (WC). The synergetic effect between CoNP and WC, as well as the high electrical conductivity of CoNP, contributed to the high activity of the prepared sensor toward hydrogen peroxide. The results exhibit a wide range of detection (50 Nm–1.02 mM) over the working voltages. The constructed sensor exhibited notable stability, as it retained 95.6% of its initial response even after a period of 30 days.

**Figure 5:**
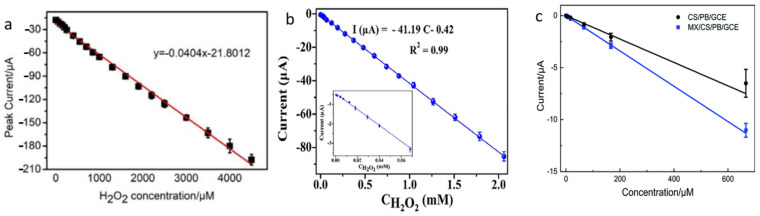
Calibration curves from amperometric response of (**a**) Mo_2_C/NC nanocomposite in the presence of 0.01 M phosphate buffer (reproduced with permission from Ref. [87], copyright 2019, Wiley), (**b**) Mo_2_C/Mo_3_P (reproduced with permission from ref. [88] copyright 2022, Elsevier), (**c**) the CS/PB/GCE (black) and MX/CS/PB/GCE (blue) in the 0.1 M phosphate buffer (pH 6.0) at an applied potential of 0 V (vs. Ag/AgCl) (reproduced with permission from Ref. [89], copyright 2021, Royal Society of Chemistry).

Mo_2_C has been extensively used for the determination of hydrogen peroxide in various conditions, including biomedical and environmental monitoring [85]. As one of the works investigating the effectiveness of Mo_2_C, a new material containing porous molybdenum carbide and nitrogen-infused carbon (p-Mo_2_C/NC) was fabricated by Li and colleagues [87]. By utilizing SiO_2_ nanocrystals as templating agents, the surface area available for reactions was significantly enhanced, thereby facilitating the precise detection of hydrogen peroxide through the integration of porous Mo_2_C, departing from the conventional technique of dispersing catalysts within porous carbon substrates. This incorporation of porous Mo_2_C introduced a novel pathway for enhancing catalytic efficiency. The outcome of the synthesized enzyme-free sensor exhibited remarkable detection prowess, prominently exemplified by an unparalleled limit of detection, notably at 0.22 μM (Figure 5a). In another work [88], a novel peanut shell-like Cu-Mo_2_C/Mo_3_P/C composite was synthesized to improve the sensitivity of the sensor toward hydrogen peroxide compared to previous studies. The prepared composite generated an exceptionally effective non-enzymatic sensor for hydrogen peroxide detection by employing a straightforward drop-coating technique onto a glassy carbon electrode (GCE), which could proficiently operate at pH 7.4. The sensor displayed (Figure 5b) a wide linear range of detection (0.55 μM to 2.06 mM), remarkable sensitivity (653.2 μA·mM^−1^·cm^−2^), and an impressively low detection limit (37 nM). The authors hypothesized that its extraordinary capabilities are due to its unique microstructure, the synergistic combination of Mo_2_C and Mo_3_P, and the inclusion of copper and carbon via dual doping. Apart from Mo_2_C, Ti_2_C_3_ was also used for hydrogen peroxide detection. For instance, Zhu et al. [89] introduced a new enzyme-free hydrogen peroxide sensor utilizing Ti_3_C_2_T_x_ and chitosan (CS) as a platform and Prussian blue (PB) as a selective electrocatalyst for hydrogen peroxide reduction. The Ti_3_C_2_/CS/PB/GCE sensor exhibited high sensitivity, a low detection limit (4 nM), and a broad linear range (50 nM–667 μM) for hydrogen peroxide detection (Figure 5c). TMCs sensors have been effectively employed in several sectors where hydrogen peroxide is used and have demonstrated high stability; nonetheless, there is still room to improve the detection limit and durability. 

### 3.2. Transition Metal Oxides

TMOs have been used as active sensing materials and are gaining popularity due to their notable features, including strong chemical stability, good biocompatibility, a wide surface area, and high electroconductivity [90]. TMOs can also be engineered to have different physical, chemical, and electrical properties, which is essential for their various applications, including biological sensing, hydrogen peroxide sensing, and gas sensing [90].

#### 3.2.1. Gas Electrochemical Sensors

Gas sensors based on TMOs have been extensively used because of their high surface area, exceptional stability, and good electrical conductivity. Many TMO-based gas sensors have been developed over the past decade, including catalytic and electrochemical types that employ distinct processes to detect gas existence. The most reported TMOs in gas sensing applications are MoO_3_, TiO_2_, and WO_3_, exhibiting short response times in practical applications, as demonstrated in Table 2 [13].

The gas detection mechanisms can be divided into microscopic and macroscopic ones. The microscopic mechanism highlights different theories, such as Fermi-level control and charge carrier depletion, while the macroscopic mechanism only illustrates the interplay between the sensor and gas. The latter method is the most common method implemented in recent works. As an example, TMO gas sensors detect the targeted gas based on the changes in their electrical properties, which are affected by the electrochemical adsorption of gas particles on the surface of TMOs. However, physical adsorption might occur on the surface of TMOs, but intermolecular forces slightly influence the electrical properties without chemical adsorption [91].

**Figure 6:**
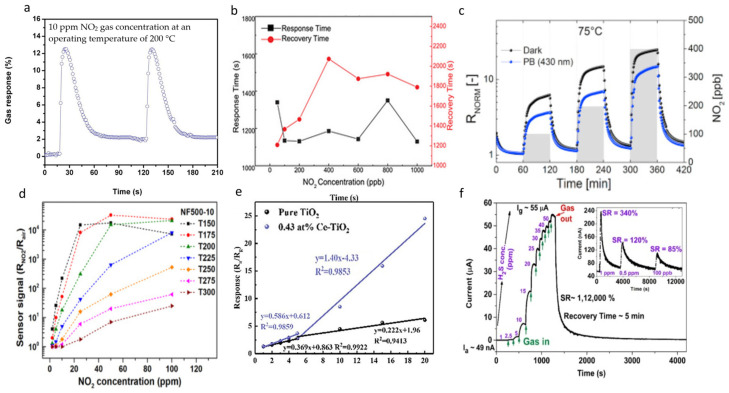
(**a**) Transient gas response curves of MoO_3_ film for two successive cycles of 10 ppm NO_2_ gas concentration at an operating temperature of 200 °C (reproduced with permission from Ref. [92], copyright 2017, Elsevier), (**b**) response and recovery time of the α-MoO_3_ sensors toward NO_2_ from 50 to 1000 ppb at 125 °C (reproduced with permission from Ref. [93], copyright 2022, Elsevier), (**c**) dynamic normalized responses of WO_3_ nanofibers to NO_2_ gas in dark conditions (black line) and purple blue light (blue line) at 25 °C (reproduced with permission from Ref. [94], copyright 2016, Elsevier), (**d**) WO_3_ nanofibers sensor signal as a function of NO_2_ concentrations at operating temperatures ranging from 150 to 300 °C (reproduced with permission from Ref. [95], copyright 2021, Elsevier), (**e**) relationship of responses values of Ce-TiO_2_ nanocrystal versus NH_3_ concentration (reproduced with permission from Ref. [96], copyright 2022, Elsevier), (**f**) response curve of nanocrystalline TiO_2_ thin film on the Si/SiO_2_ substrate (reproduced with permission from Ref. [97] copyright 2021, Elsevier).

MoO_3_ with different morphologies, including needle, nanorod, and plate, has been fabricated and tested as a gas sensor. For instance, Mane and Moholkar [92] deposited nanobelt-like MoO_3_ onto the glass substrate to detect NO_2_ gas. They obtained the greatest response of 68% for NO_2_ gas at 200 °C, while the lower detection limit was discovered to be 10 ppm with a reaction time of 15 s, which may serve as a safe sensor but still has to be improved at lower temperatures (Figure 6a). The MoO_3_ nanoribbons synthesized by Li et al. [93] exhibited an exceptional detection limit of 24 ppb toward NO_2_ at 125 °C (Figure 6b). Despite their excellent selectivity and sensitivity to NO_2_, their produced sensor had a prolonged response and recovery time. 

Several studies have reported WO_3_ with different morphologies as a good detector of NO_2_ gas, which all illustrated high selectivity and sensitivity toward NO_2_ [98,99]. This investigation indicated that the selectivity and the detection limit of the NO_2_ gas sensor are dependent on WO_3_ morphology. Among WO_3_ with different morphologies, nanofiber WO_3_ has been studied more extensively than others because of its higher sensitivity. For instance, Giancaterini et al. [94] prepared a room-temperature NO_2_ sensor made of WO_3_ nanofibers. They examined the sensitivity of the sensor toward NO_2_ under various realistic conditions, such as visible light illumination. Figure 6c displays the electrical response of the sensor in the presence of purple-blue light illumination at 75 °C, which leads to the conclusion of the positive effect on recovery percentage and baseline resistance. In a similar study, Morais et al. [95] used electrospinning to synthesize nanofiber WO_3_ that could work in wide working temperatures up to 300 °C. Figure 6d represents the ratio of sensor signals from NO_2_ to air in different NO_2_ concentrations and operating temperatures. Their findings pointed out that their sensor exhibited the maximum sensitivity to NO_2_ gas at 150 °C, which is in the presence of 25 ppm NO_2_. However, NO_2_ is not the only gas that the WO_3_ sensor can sense. As an example, a hierarchical WO_3_/CuO sensor was fabricated to sense the xylene gas, a typical VOC [100]. The prepared WO_3_/CuO showed a fast and high response to xylene gas in addition to its long-term stability, which is caused by the construction of p-n junctions in the hierarchical structure. 

TiO_2_ is another TMO that has been fabricated to sense different gas species, encompassing hydrogen sulfate (H_2_S) and NH_3_ [96,97]. TiO_2_ nanocrystal is one of the types of TiO_2_ that has been exploited as a gas sensor. Wu et al. [96] fabricated small-size Ce-TiO_2_ nanocrystals with exceptional crystallinity and a large surface area to detect NH_3_ gas. They demonstrated a selective and repeatable sensor with a low detection limit (140 ppb) and high response (23.99 at 20 ppm) (Figure 6e). The high sensitivity and selectivity of their fabricated sensor were explained by a synergetic strategy that involved the re-orientation of high-energy facets, the presence of oxygen vacancies, and a large specific surface area, which was also proved by their DFT calculations. TiO_2_ not only showed high sensitivity and long-term stability toward NH_3_ sensing but also exhibited a reliable sensor for H_2_S gas. Nanocrystalline TiO_2_ also displayed a quick response toward H_2_S, as low as 48s, in a study conducted by Nagmani et al. [97]. As depicted in Figure 6f, the lowest limit of the H_2_S detection was 100 ppb, which was caused by high surface roughness and defects. While many sensitive gas detectors have been prepared, the versatility of the sensor in various conditions, such as high temperature, needs further improvement to be used in practical applications. 

**Table 2:** Comparison of analytical parameters of TMC and TMO electrodes for gas sensing applications.

Ref.	Electrode	Analyte	Response Ra/Rg	LOD	Response/Recover Time
[96]	Ce-TiO_2_	NH_3_	23.99	140 ppb	55 s/192 s
[97]	TiO_2_	H_2_S	1120	100 ppb	48 s/5 min
[91]	WO_3_	NO_2_	12.4	400 ppb	-
[95]	WO_3_	NO_2_	-	25 ppm	15/0.8 min
[97]	WO_3_/CuO	xylene	-	15 ppm	5.5/17.5 s
[92]	MoO_3_	NO_2_	68	10 ppm	15/150 s
[93]	MoO_3_	NO_2_	0.05	24 ppb	-
[66]	Ti_3_C_2_T_x_	NH_3_	0.21	9.27 ppm	-
[68]	Ti_3_C_2_T_x_	Acetone	0.97	50 ppb	-
		Ethanol	1.7	100 ppb	-
		NH_3_	0.8	100 ppb	-
[72]	Nb_2_CT_x_-CTAB	NO_2_	0.66	21 ppb	55 s/60 s
[69]	Ti_3_C_2_T_x_/graphene	NH_3_	0.94	10 ppm	-
[73]	V_2_CTx	Acetone	0.978	11.2 ppm	-
		Methane	0.983	9.4 ppm	8 min/5.5 min
		H_2_	0.804	1.4 ppm	2 min/7 min
		H_2_S	0.995	3.5 ppm	-

#### 3.2.2. Biological Electrochemical Sensors

**Figure 7:**
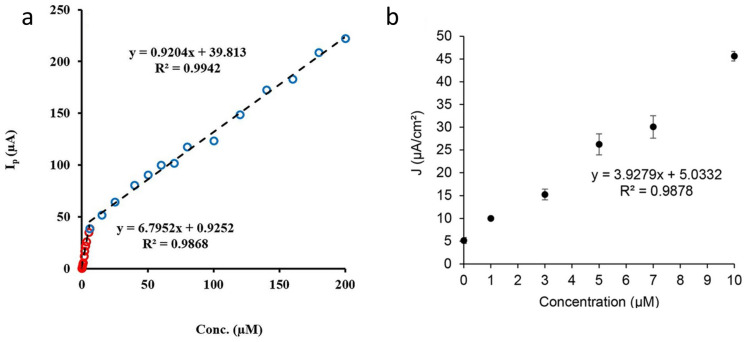
The calibration curve of (**a**) Fe_3_O_4_@PPy in a Britton Robinson solution (pH = 8) for MET detection (reproduced with permission from ref. [105], copyright 2021, Elsevier). (**b**) DPV measurements of dopamine from 0 to 10 μM via GO/Fe3O4 electrode (reproduced with permission from Ref. [104], copyright 2022, Taylor and Francis).

TMOs-based biosensing is progressive due to the appropriate electrocatalytic, good electrical properties, and biocompatibility of TMOs [101]. Iron oxide and titanium dioxide are the most renowned biocompatible TMOs, which are implemented in immobilizing biomolecules to fabricate immunosensors, DNA, and enzyme sensors [102,103,104]. Riahifar and colleagues [105] developed a new sensor to detect methamphetamine (MET), a drug that causes severe and irreversible damage to the nervous system. This sensor is designed using a core-shell structure called Fe_3_O_4_@PPy. The prepared sensor showed a linear response across a range of 0.005 to 200 μM, as shown in Figure 7a. This MET sensor showed long-term stability and also had an incredibly low detection limit, as low as 1 nM. Fe_3_O_4_ is also beneficial for the determination of secreted hormones like dopamine, which is crucial because any abnormalities can cause disease. In a recent study, it was indicated that the combination of Fe_3_O_4_ and graphene oxide (GO) is able to selectively detect dopamine [104]. The CV results suggested that GO can effectively play a role in enhancing surface area and conductivity, resulting in a decreased detection limit (0.48 μM). The DPV for the GO/Fe_3_O_4_ sensor demonstrated a linear response in the range of 1 to 10 μM (Figure 7b). In one of the newest applications of Fe_3_O_4_ as a biosensor, Fe_3_O_4_ combined with gold nanoparticles and reduced GO (rGO) to detect miRNA-128, a biomarker of lymphoblastic leukemia [106]. The redox mediators employed in this study were hexacyanoferrate and methylene blue, which played a crucial role in enhancing the ability of the sensor to detect the targeted biomolecule even at low potentials in a phosphate buffer saline solution, hence improving the accuracy of the detection process. However, the result indicates that the detection limit of methylene blue (0.005483 fM) is higher than that of (0.05346 fM) hexacyanoferrate. 

Apart from Fe_3_O_4_, several research papers have extensively investigated the potential of TiO_2_-based sensors as electrochemical biosensors [107,108,109]. Cancer detection is one of the most attractive applications of TiO_2_-based materials because of their simple mechanism, high accuracy, and good selectivity of biomarker detection. Jalil et al. [102] prepared a nanocomposite made of rGO and TiO_2_ nanoparticles aiming for the early detection of epithelial cell adhesion molecules (EpCAM), a tumor biomarker. DPV with the potential range of −0.4 to 0.8 V and amplitude of 50 mV showed a low detection limit (0.0065 ng·mL^−1^) and a wide detection range (0.01 ng·mL^−1^ to 60 ng·mL^−1^). TiO_2_-based sensors with different morphologies were also constructed for tumor diagnosis. A label-free detector for cancer antigen 125 (CA125) was fabricated utilizing TiO_2_ nanotube, gold, and vertical graphene (VG) [110]. TiO_2_ nanotubes were synthesized via CVD, and Au nanoparticles were incorporated into the root and surface of the vertical graphene. They reported that the response range of the Au-Graphene/TiO_2_ was between 0.01 and 1000 mU∙mL^−1^ in human serum, in which the sensor recovery reached up to 99.8%. Table 3 represents a comparison between the TMO and TMC electrochemical sensors performance in biological applications. 

#### 3.2.3. Hydrogen Peroxide Electrochemical Sensors

The utilization of TMOs in electrochemical sensing of hydrogen peroxide is increasingly preferred compared to conventional techniques, such as fluorometry, spectrometry, and chromatography, primarily due to its cost effectiveness and simplicity [116,117]. TMOs (e.g., TiO_2_, Fe_3_O_4_, and ZnO) have shown good promise in this regard. Integrating Fe_3_O_4_ nanoparticles with graphene supported by carbon cloth was used to detect hydrogen peroxide [118]. This flexible sensor illustrated a satisfactory detection limit as low as 4.79 μM, with an optimized linear range between 10 and 110 μM. The sensor exhibited a high level of stability, as it demonstrated a 94.7% recovery of its initial current after 10 days. In a similar work, a non-enzymatic hydrogen peroxide sensor was made of Fe_3_O_4_, rGO, and nickel (Ni) [119]. Enhancing charge mobility was achieved by lowering the band gap of the modified Fe_3_O_4_ with Ni, inducing a low detection limit of 0.2 μM. Moreover, the prepared sensor exhibited a wide linear range (1 to 1000 μM), high sensitivity (6012 mA·M^−1^), and good selectivity toward hydrogen peroxide.

Furthermore, TiO_2_ nanoparticles served as the active material for a hydrogen peroxide sensor, as they indicated a strong affinity for the chemisorption of hydrogen peroxide on their surface. In the investigation of Rathinam et al. [120], it was revealed that TiO_2_ nanoparticles exhibited the capability to detect hydrogen peroxide within a concentration range spanning from 0.1 to 50 mM. However, their reported detection limit of 0.061 mM was comparable to other hydrogen peroxide sensors listed in Table 4. Meanwhile, Karatekin [121] attempted to boost the sensitivity and detection limit of hydrogen peroxide by leveraging the synergistic effects of incorporating TiO_2_ nanoparticles with N and S-doped rGO. Their findings also demonstrated that incorporating S atoms into GO layers led to a significant enhancement in the catalytic activity of the sensor, hence improving its sensitivity towards hydrogen peroxide. Consequently, the prepared sensor exhibited a linear range, sensitivity, and detection limit of 2 to 1000 μM, 188.75 µA·mM^−1^, and 0.019 µM, respectively. In summary, electrochemical sensing of hydrogen peroxide with TMOs is gaining attention. Nevertheless, it is imperative to enhance the sensitivity and detection limit of the sensor in order to guarantee its efficacy, given the relatively low concentration of hydrogen peroxide in the organs. 

Based on the data shown in Table 2, Table 3 and Table 4, it can be inferred that TMC electrochemical sensors have a wider linear range, making them more versatile compared to TMO electrochemical sensors. In addition, TMCs exhibit a lower LOD in biological and hydrogen peroxide sensing applications, indicating their heightened sensitivity. However, TMOs exhibited better performance than TMCs in electrochemical gas sensing due to their more efficient charge transport and broader bandgaps.

## 4. Summary and Future Outlook

TMOs and TMCs have captured tremendous attention in sensing applications due to their outstanding features, such as varied functionalization, good chemical and physical stability, large surface area, strong oxidation ability, and tunable electronic characteristics. The cost-effective production of electrochemical sensors based on TMOs and TMCs presents several advantages over chromatography devices, optical fibers, and chemiresistive devices. These advantages include enhanced sensitivity and selectivity, improved adaptability, and reduced complexity, rendering TMOs and TMCs sensors more appealing in various applications. Several techniques have been employed to manufacture TMCs and TMOs, with the hydrothermal process being the most utilized technique, utilizing some hazardous materials (e.g., HF, chlorine gas, etc.). However, this method led to severe environmental issues due to exploiting hazardous materials, and so CVD-assisted methods, rapid annealing, etc. have been used instead. Regardless of the fabrication methods, fabricated TMCs and TMOs are widely used for gas sensors, biosensors, and hydrogen peroxide sensors. However, some obstacles still exist for the commercialization of these sensors:

- TMOs and TMCs sensors have not shown good stability at low temperatures (<30 °C) because of their strong oxidation ability. Although the strong oxidation affinity of TMCs and TMOs is good for sensitivity, it might cause serious problems for the stability of the sensors at room temperature, which can be resolved by using different techniques, such as chemical doping and benefiting from composite materials.
- The necessity for rapid reactions is crucial in several applications. However, the sensors stated earlier demonstrated extended response times, indicating a need for additional improvement. This enhancement may be accomplished by optimizing the size, dimension, and material composition of the sensors.
- The manufactured sensors exhibited enhanced durability during extended periods of operation. However, in order to optimize their suitability for industrial applications, it is imperative to further enhance the lifespan of these sensors, particularly for their operation in extreme environmental circumstances.
- It is worthwhile to mention that TMO electrochemical sensor performance can be influenced by capping agents, molecules that are attached to the surface of nanoparticles to prevent them from agglomeration, as they have been used in TMO synthesizing [122]. One of the potential impacts of capping agents might be an improvement in the selectivity and sensitivity of the TMO sensors. This would be due to the fact that capping agents exert an influence on the electronic structure of TMOs, which can change the surface of the sensors bonding to the targeted molecule [123]. Another impact of using capping agents could be reducing the toxicity of the sensors, which is an important parameter, especially in biosensing applications [124]. Capping agents have the potential to enhance the structural and chemical stability of sensors, which can ultimately result in improved stability of sensing performance [122,124]. However, the extent of capping agent influence on the performance of the TMO electrochemical sensors needs further investigation.
- When operating under high potentials in alkaline environments, the surfaces of TMOs and TMCs may be oxidized and transformed to (oxy)hydroxides [125,126]. Consequently, the coordination and electronic properties of the parent metal will be changed by the introduction of these heteroatoms as interstitials or substitutes. The effect of such surface transformations on sensing performance (e.g., sensitivity, detection limit, and long-term stability) should be further investigated.

## Figures and Tables

**Table 1:** An overview of advantages and disadvantages of TMOs and TMCs fabrication methods.

Synthesis Method	Advantage	Disadvantage
Chemical Vapor Deposition (CVD) Method	**High Purity**: Capable of producing materials with high purity levels. **Uniformity**: Offers high uniformity in film thickness and composition. **Precision Control**: Allows precise control over film thickness and composition through parameter adjustment.	**High Cost**: Associated with higher equipment and operational costs. **Temperature Constraints**: Requires high temperatures, potentially unsuitable for thermally sensitive materials. **Complexity**: The process is relatively complex and requires meticulous operation.
Hydrothermal Method	**Low-Temperature Synthesis**: Generally, operates at lower temperatures. **Environmentally Friendly**: Utilizes water as a solvent, minimizing environmental impact. **Crystallinity**: Capable of producing high-quality crystals.	**Long Reaction Times**: Typically requires longer durations to complete reactions. **Size Control Challenges**: Control over particle size and shape is relatively difficult. **Scalability Limitations**: Limited scalability for large-scale production.
Controlled Electrodeposition Method	**Precise Control**: Enables precise control over the thickness and composition of the deposited material. **Cost Effectiveness**: Generally lower in equipment and operational costs compared to other methods. **Low-Temperature Operation**: Conducted at room temperature or lower, making it suitable for thermally sensitive materials. Versatility: Applicable to a wide range of materials, including nanomaterials.	**Uniformity Issues**: Sometimes challenging to ensure uniform deposition layers. **Scale Limitations**: Challenges in achieving deposition over large areas. **Electrochemical Complexity**: Involves complex electrochemical processes, requiring skilled operation.
Acid Etching Method	**High Precision**: Allows for accurate control of etch depth and shape. **Selectivity**: Specific materials can be targeted for etching by choosing appropriate acids. **Wide Applicability**: Usable on a variety of materials.	**Hazardous**: Hydrofluoric acid is extremely dangerous, necessitating stringent safety measures. **Environmental Impact**: environmental pollution. **Control Complexity**: The etching process can be complex to control, requiring meticulous adjustment.

**Table 3:** Comparison of analytical parameters of TMC and TMO electrodes for biological sensing applications.

Ref.	Electrode	Analyte	Sensitivity	LOD	Linear Range
[111]	Iron oxide	Glucose	5.31 μA·mM^−1^·cm^−2^	7 μM	0.25–8 mM
[103]	TiO_2_/Au	Ascorbic acid	35,900 μA·mM^−1^·cm^−2^	1.2 µM	5–51 µM
[112]	TiO_2_/rGO	Glucose	35.8 μA·mM^−1^·cm^−2^	4.8 µM	0.032–1.67 mM
[113]	Fe_3_O_4_@Ag/CPE	Olanzapine	0.50 µA·mM^−1^	0.0018 µM	0.39–38.4 µM
[114]	Fe_3_O_4_/GO	Dopamine uric acid	0.12 µA·mM^−1^	0.053 µM	0.1–150 µM
[102]	rGO/TiO_2_	Epithelial cell adhesion molecules	3.24 µA·ng^−1^·mL·cm^−2^	0.0065 ng·mL^−1^	0.01–60 ng·mL^−1^
[104]	Fe_3_O_4_/GO	Dopamine	-	0.48 µM	1–10 µM
[110]	Au–VG/TiO_2_	Cancer antigen 125	14.82 μA·(log(mU·mL^−1^))^−1^	0.0001 mU·mL^−1^	0.01–1000 mU·mL^−1^
[115]	Ti_3_C_2_T_x_	Glucose	35.3 μA·mM^−1^·cm^−2^	0.33 μM	10–1500 μM
		Lactate	11.4 μA·mM^−1^·cm^−2^	0.67 μM	10–22,000 μM
[81]	*f*–Ti_3_C_2_	CEA	37.9 µA·ng^−1^·mL·cm^−2^	18 fg/mL	100 fg/mL^−2^ μg/mL
[78]	MoS_2_/Ti_3_C_2_	miRNA-182	-	0.43 fM	1 fM to 0.1 nM
[79]	GSH-MQDs	miRNA-221	-	10 fM	10 fM to 10 nM

**Table 4:** Comparison of analytical parameters of TMC and TMO electrode for H_2_O_2_ sensing applications.

Ref.	Electrode	Sensitivity	Detection Limit	Linear Range
[118]	Fe_3_O_4_/Graphene	0.037 µA·μM^−1^·cm^−2^	4.79 μM	10 to 110 μM
[119]	Ni–Fe_3_O_4_@s-rGO	6.012 µA·μM^−1^	0.2 μM	1 to 1000 μM
[120]	TiO_2_ nano-particles	N/A	0.061 mM	0.1 to 50 mM
[121]	TiO_2_@NS-rGO	0.188 µA·μM^−1^	0.019 µM	2 to 1000 μM
[86]	WC/CoNP	6.696 μA·μM^−1^·cm^−2^	6.3 nM	50 nM to 1.0 mM
[87]	p-Mo_2_C/NC	0.577 μA·μM^−1^·cm^−2^	0.22 μM	0.05 to 4.5 mM
[88]	Cu-Mo_2_C/Mo_3_P/C	0.653 μA·μM^−1^·cm^−2^	37 nM	0.55 μM to 2.06 mM
[89]	MX/CS/PB/GCE	N/A	4 nM	50 nM to 667 μM

## Data Availability

No data available.
